# Vegetation in clear-cuts depends on previous land use: a century-old grassland legacy

**DOI:** 10.1002/ece3.1288

**Published:** 2014-10-24

**Authors:** Dennis Jonason, Mathias Ibbe, Per Milberg, Albert Tunér, Lars Westerberg, Karl-Olof Bergman

**Affiliations:** IFM Biology, Conservation Ecology Group, Linköping UniversityLinköping, SE-581 83, Sweden

**Keywords:** Extinction debt, habitat fragmentation, historical maps, plant traits, production forestry, remnant populations, seminatural grasslands

## Abstract

Plant species richness in central and northern European seminatural grasslands is often more closely linked to past than present habitat configuration, which is indicative of an extinction debt. In this study, we investigate whether signs of historical grassland management can be found in clear-cuts after at least 80 years as coniferous production forest by comparing floras between clear-cuts with a history as meadow and as forest in the 1870s in Sweden. Study sites were selected using old land-use maps and data on present-day clear-cuts. Species traits reflecting high capacities for dispersal and persistence were used to explain any possible links between the plants and the historical land use. Clear-cuts that were formerly meadow had, on average, 36% higher species richness and 35% higher richness of grassland indicator species, as well as a larger overall seed mass and lower anemochory, compared to clear-cuts with history as forest. We suggest that the plants in former meadows never disappeared after afforestation but survived as remnant populations. Many contemporary forests in Sweden were managed as grasslands in the 1800s. As conservation of remaining grassland fragments will not be enough to reduce the existing extinction debts of the flora, these young forests offer opportunities for grassland restoration at large scales. Our study supports the concept of remnant populations and highlights the importance of considering historical land use for understanding the distribution of grassland plant species in fragmented landscapes, as well as for policy-making and conservation.

## Introduction

The effects of previous land use on ecosystems may linger for a long time after the use has ceased (Plue et al. 2008). Knowledge of historical land use and recognition of its legacies in ecological systems may therefore add explanatory power to the interpretation of current patterns of species distribution and abundance and help guide policymakers and conservationists in their decision making (Foster et al. 2003).

Owing to the region's long history of traditional management of grazing and hay-making, seminatural grasslands (i.e., unfertilized pastures and meadows) in central and northern Europe possess a highly unique and species-rich flora (Kull and Zobel 1991; Eriksson et al. 2002). The small-scale plant richness can be exceptionally high, with over 60 species per square meter (Kull and Zobel 1991). However, drastic land-use changes over the last century have caused most seminatural grasslands to disappear through either plowing (including fertilization), afforestation, or abandonment (Dahlström et al. 2006). In several countries, the reduction in total area has been close to 100% (Luoto et al. 2003; Polus et al. 2007; Cousins and Eriksson 2008; Hooftman and Bullock 2012). Despite the significant habitat loss, the number of grassland plant species has not declined to a similar extent (e.g., Lindborg and Eriksson 2004). This may be the result of an extinction debt (Tilman et al. 1994), that is, delayed extinction following a forcing event. Several studies have found that present-day plant species richness in seminatural grasslands is more closely linked to past than current landscape composition (Lindborg and Eriksson 2004; Helm et al. 2006; Krauss et al. 2010). Although plant species are destined to go extinct in the remaining fragments, there is still an opportunity to prevent further species loss through habitat restoration and conservation efforts (Kuussaari et al. 2009).

In Sweden, a large proportion of the former seminatural grasslands on poor soils are currently managed as coniferous production forests (Cousins 2009). Given the possibility that plants can resist extinction by surviving as remnant populations (Eriksson 1996; Johansson et al. 2011) and the occurrence of persistent soil seed banks (Milberg 1995), it is possible that these forests contain signs of their historical land use in the flora. If so, this would offer novel opportunities for grassland restoration and conservation at large scales. Previous studies have examined remnant plant populations after abandonment or restoration of seminatural grasslands (Pykälä 2003; Johansson et al. 2011), but how plants respond to a complete change in land use, from grassland to coniferous production forest, is fairly unknown (but see Bisteau and Mahy 2005).

The aim of this study was to investigate whether the flora of newly created clear-cuts, after a full generation of coniferous production forest, can show signs of historical grassland management. We compared the floras of clear-cuts with a history as meadow in the 1870s to those of clear-cuts that were forested at that time. The investigated sites were selected with the help of old regional maps showing land use (“Häradsekonomiska kartan”) overlaid with recent maps of clear-cuts. To discern any possible affiliation of the plants to the two clear-cut categories, species traits reflecting high capacities for dispersal and persistence were analyzed. We hypothesized that we would find grassland plant species in clear-cuts previously managed as meadow and established three possible scenarios as explanations: (1) The grassland plants never disappeared after afforestation but have survived as remnant populations, (2) the plants arrived from neighboring grasslands after logging, and (3) the plants have persisted in the soil seed bank and emerged after clear-cutting. Remnant populations are associated with persistence-related traits such as perennial life-form, large seed mass, and low dispersal ability (Eriksson 1996; Johansson et al. 2011), and dominance of such traits in clear-cuts with a history as meadow was predicted to support the first scenario. Furthermore, dominance of short-lived species and species with high dispersal ability, as well as species with persistent seed bank, was predicted to support the second and third scenario, respectively.

## Materials and Methods

### Study area and site selection

The study was conducted in the province of Östergötland, in the south of Sweden (N57° 43′–58° 15′; E15° 00′–15° 40′). The landscape has a history of extensive agriculture, but is today largely dominated by coniferous production forests.

Forty-eight clear-cuts were selected for the study, 24 in each of the years 2009 and 2013. Half of the clear-cuts in each year were managed as either meadow or forest in the 1870s. The categorization was made by cross-referencing a land-use map from the 1870s with data on clear-cuts from the Swedish Forest Agency (to which landowners have to report planned clear-cut operations). Old land-use maps are often the only source of information on how the landscape was composed at that time and show features such as arable fields, coniferous and deciduous forests, meadows, wetlands, and roads. For a clear-cut to be categorized as former meadow, the meadow would have to cover a minimum of 15% of its area. We used the whole clear-cut as the unit for our study and not only the part that was formerly meadow, both because of the relatively low accuracy of old maps and because the clear-cuts will be the units in a future study directed at forest managers focusing on applications of the results in practice. Exactly when the meadows were converted into forest is not known, but all sites have hosted at least one generation of coniferous production forest, corresponding to approximately 80–120 years. For clear-cuts categorized as having a forest history, we cannot exclude that some could have been meadow prior to the 1870s when the maps were created. This is, however, unlikely given that the largest transition from meadow to forest begun at least two decades later (Kardell 2004).

After the cessation of meadow management, some sites may initially have been grazed by livestock, a practice that became rare and eventually ceased in the 1930s due to demands for increased forest productivity (Kardell 2004). Moreover, in the 1800s, high grading (i.e., selective cutting of the most merchantable trees) was the principal harvesting method and clear-cutting rarely occurred. This pattern of forest management, together with forest grazing and frequent fires, sustained multiaged and semiopen stands with small treeless openings (Niklasson and Granström 2000; Axelsson and Östlund 2001). However, in line with the introduction of modern forest management around the 1950s, clear-cutting took over as the general harvesting method and the forests progressed into dense and uniformed production forests.

The size of the clear-cuts varied from 1.5 to 6.6 ha, and they had been logged 2–4 years prior to the field work (Table 1). The size limitation was chosen for practical reasons and to reduce the influence of species-to-area relationships, and the time interval was chosen because grasses and herbs are the dominant species during this time (Palviainen et al. 2005); therefore, it is the most likely period during which remnant species from seminatural grasslands might appear. To reduce the influence of nearby plant-rich habitats on the clear-cut flora, all clear-cuts were located a minimum of 300 m from any present-day seminatural grassland subjected to agri-environment support. With the plants at stake, this distance is well beyond the average dispersal distance of seeds (Thomson et al. 2011) and we therefore consider sites as carrying separate populations. Similarly, the distance between any two clear-cuts was >300 m.

**Table 1:** Comparison of site characteristic variables (mean ± 95% CI) between clear-cuts historically managed as meadow and as forest. The basal area of coniferous trees and total basal area of trees differed between clear-cut categories to a degree corresponding to *P* < 0.05

	History as meadow		History as forest	
Variable	Mean	CI 95%	Mean	CI 95%
Size (ha)	3.9	3.31–4.55	3.2	2.77–3.65
Time since clear-cutting (year)	5.0	4.17–5.83	4.8	3.85–5.74
Area historically managed as meadow (ha)	1.8	1.31–2.34	–	–
Connectivity to seminatural grasslands	106.4	77.38–135.47	86.3	58.67–113.92
Basal area coniferous trees (m^2^·ha^−1^)	41.0	37.49–44.53	35.9	33.64–38.23
Basal area deciduous trees (m^2^·ha^−1^)	1.2	0.75–1.57	0.8	0.46–1.18
Basal area total (m^2^·ha^−1^)	42.2	38.70–45.64	36.8	34.37–39.14
Logging residues (%)	2.9	1.84–4.00	4.1	2.61–5.67
Exposed mineral soil (%)	0.6	0.13–0.98	0.9	0.34–1.46
Bare rock (%)	4.2	2.93–5.44	5.6	4.03–7.25

### Plant survey

The species richness of herbs and grasses (hereafter referred to as plants) was surveyed once between August and early October. This late inventory means an under-representation in the data of some species with an early growth peak and early senescence of leaves (Bergfur et al. 2004), but was expected not to affect the overall results. One-hundred circular sample plots (radius 1 m) were evenly distributed over each clear-cut along straight transects placed 25 m apart. In the circles, presence–absence data were recorded, and species frequency was calculated as the number of sample plots with presence. Species that could not with certainty be identified to species level, predominantly belonging to the family Poaceae and the genus *Carex*, were left out. *Melampyrum pratense* and *M. sylvaticum* were difficult to distinguish when the field work was conducted and were treated together. A classification of the plants as indicators of Swedish seminatural grasslands was made after Ekstam and Forshed (1992) and Bertilsson and Paltto (2003). Species identification and nomenclature follows Karlsson (1998).

### Plant traits

To explore the relationship between species traits and the plants' responses to land-use history, six traits hypothesized to affect species dispersal and persistence (Eriksson 1996; Fischer and Stöcklin 1997; Stöcklin and Fischer 1999; Ozinga et al. 2007; Johansson et al. 2011) were selected for analysis: (1) life span, categorized as perennial or annual/biennial; (2) diaspore mass, including all appendages (henceforth seed mass); (3) seed bank persistence, categorized as short term (<25 years) or permanent (>25 years); (4) anemochory (wind dispersal), a ranking index based on the terminal velocity of a falling diaspore (i.e., the maximum velocity in still air) of c. 2700 European plant species, where a high value indicates low dispersal by wind; (5) grazing tolerance, that is, the extent to which the plants are adapted to grazing and mowing, categorized as high, neutral, or low; and (6) light tolerance based on the Ellenberg indicator value for light, categorized as high, neutral, or low. The log of diaspore mass and the anemochory index were negatively correlated (Pearson's *r* = −0.59). This was expected because heavier seeds generally have higher terminal velocities, making long-distance dispersal by wind less likely. However, both traits were kept for analysis, as they were used as measures of different survival strategies. No other traits were correlated in this data set.

Due to a lack of data, not all traits could be assigned to all species in the study, but each trait could be assigned to at least 72% (range 72–100%) of all species. Trait data were taken from the LEDA Trait base (Kleyer et al. 2008), the Dispersal and Diaspore Database (Hintze et al. 2013), and a database on traits of plants in southern Sweden (http://www.lundsbotaniska.se) (Appendix S1).

### Habitat factors

At every third sample plot in the plant survey, the species identity and diameter of stumps, snags, and living trees >10 cm in diameter were recorded within an area of 100 m^2^ (circle with radius 5.64 m). These values were then converted to basal area to obtain an estimate of the tree composition and density before clear-cutting. Additionally, within the same sample plots, the percent cover of bare rock, logging residues, and exposed mineral soil was assessed by eye. This assessment was performed to ensure that there were no systematic differences among the study sites (Table 1), as rocks and logging residues may limit the available growing-space, and exposed mineral soil may provide important opportunities for the colonization and germination of seeds.

Present-day connectivity (*C*_*i*_) to neighboring seminatural grasslands was calculated using the incidence function model (Hanski 1994; Moilanen and Nieminen 2002):


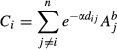


where *α* is a parameter of the negative exponential dispersal kernel scaling the effect of distance on connectivity, *d*_*ij*_ is the border-to-border distance (km) between clear-cut *i* and grassland *j*, *A*_*j*_ is the area (ha) of grassland *j*, and *b* is a scaling parameter of dispersal from *A*_*j*_. The parameter *α* was set to one after (Lindborg and Eriksson 2004), and *b* was set to 0.3 based on the recommendations in (Moilanen and Nieminen 2002). Hence, the formula defines connectivity as the total area of neighboring seminatural grasslands weighted by the distance of each grassland patch to respective clear-cuts. Spatially explicit data on seminatural grasslands were extracted from a geographical database provided by the Swedish Board of Agriculture (TUVA database; http://www.sjv.se/tuva).

### Statistical analyses

To investigate whether there were any systematic differences between the two clear-cut categories that could potentially affect the interpretation of the result, the mean and 95% confidence interval (CI 95%) for 10 site characteristic variables were compared (Table 1). Adjoining CIs are equivalent to *P* = 0.01 and overlap by a maximum of half an error bar to *P* = 0.05 (Cumming et al. 2007).

The assessment of the plant species' preferences for one of the two categories of clear-cuts involved two steps, both using random effect meta-analysis methods within the software Comprehensive Meta-Analysis (Borenstein et al. 2005). First, a logit-transformed event rate (±SE) for each species on each clear-cut was calculated based on plant frequency and sample size, for example, if a species was found in 32 out of 100 sample plots, it gave an event rate (ER) of 32/100 and logit(ER) = log(ER/(1-ER)) for that clear-cut. These estimates of clear-cut frequencies were then subjected to a meta-analysis calculating an overall estimate for all 24 clear-cuts with the same history, resulting in two values per species. The difference between these values (i.e., Δ logit(ER)) and the added variances were used in the second step of the meta-analysis calculating effect sizes with CI 95% where a positive sign indicated a preference for clear-cuts previously managed as meadow. Only species that occurred on at least two clear-cuts were included in this meta-analysis to reduce the bias from giving too much weight to rare species. Meta-regression analyses were used to examine the effect of species' traits on effect sizes. To reduce the influence of the large variation in seed mass, that trait was log-transformed prior to analyses. All other variables were kept untransformed.

## Results

**Figure 1:**
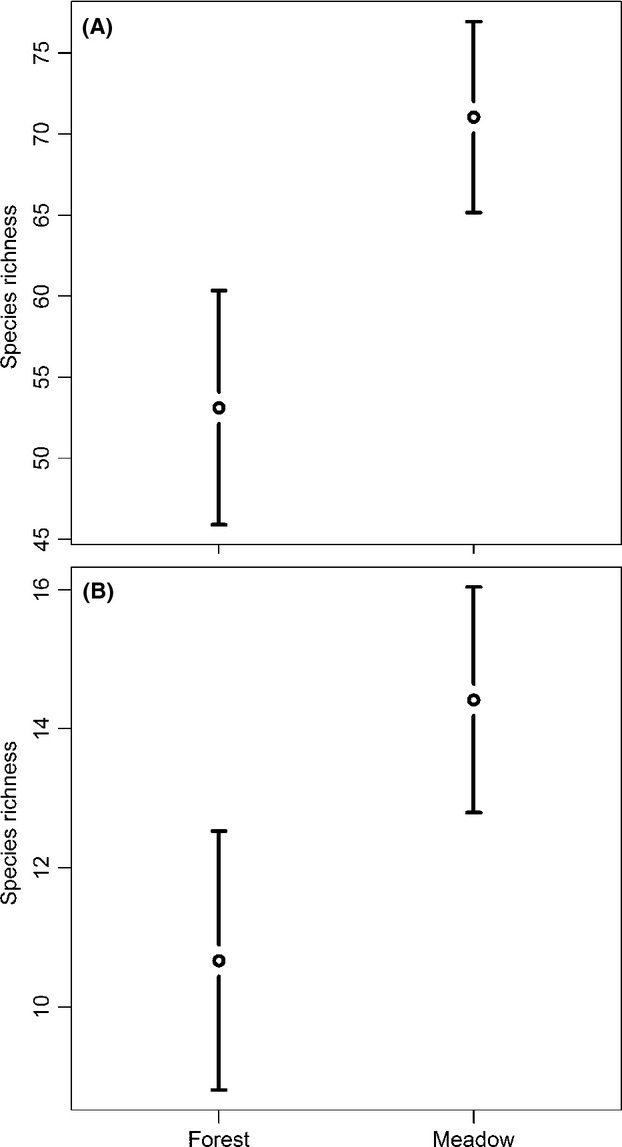
Average species richness with CI 95% of (A) all plants and of (B) plants classified as grassland indicator species in clear-cuts historically managed as forest and as meadow.

In total, 179 plant species were found in the study, of which 37 were indicators of seminatural grasslands (Appendix S2). Clear-cuts with a history as meadow had, on average, 36% higher species richness compared to clear-cuts with a long history as forest (Fig. 1A) and a 35% higher richness of grassland indicator species (Fig. 1B), although the total number of species found was fairly similar between the clear-cut categories (Appendix S2).

The basal area of coniferous trees as well as the total basal area of trees was higher before logging on clear-cuts with a history as meadow (Table 1). This difference can be considered a consequence of land-use history, as hay meadows were generally situated on productive soil types, whereas less productive soils were allocated to forest production (Dahlström et al. 2006). Thus, those variables were not treated as covariables in the statistical analyses. No other environmental or site characteristic variable differed between the two clear-cut categories (Table 1).

**Figure 2:**
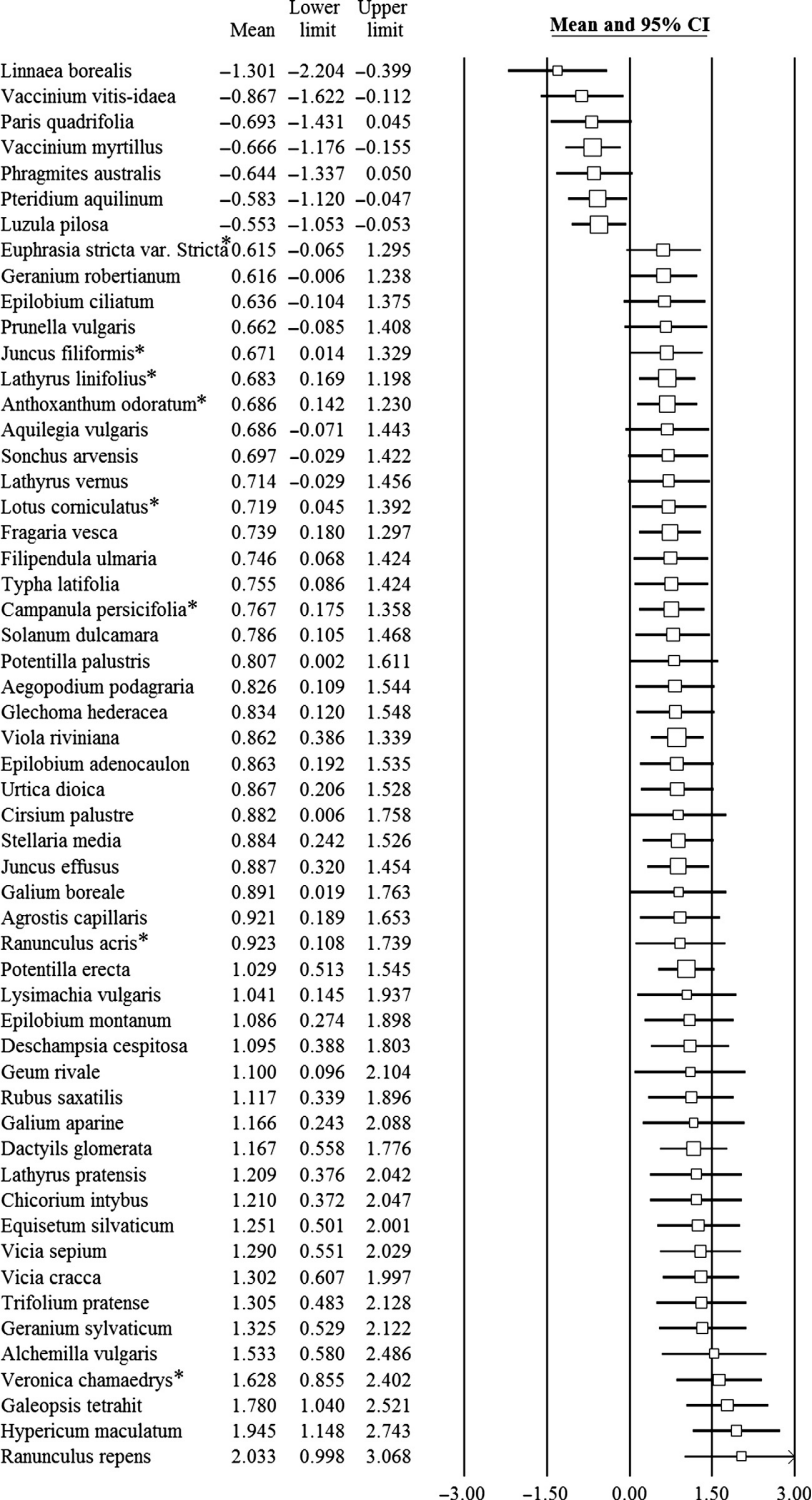
Forest plot of plant species' preferences for clear-cuts with history as meadow (positive values) or as forest (negative values). Only species overlapping zero with >0.1 are shown. Species denoted by a “*” are classified as grassland indicators.

The average effect size returned from the meta-analysis of 154 species (i.e., those occurring on >1 site) provided clear support for an effect of historical grassland management on plant species richness (weighted average effect size 0.36, CI 95% 0.27–0.45). A total of 42 species were significantly confined to clear-cuts with a meadow history compared to only five species for clear-cuts with a forest history (Fig. 2). An analysis including only the indicator species also gave strong support for this pattern (weighted average effect size 0.37, CI 95% 0.21–0.52). Seven of the 33 indicator species included in the analyses were significantly associated with clear-cuts that were previously meadow and none with clear-cuts with a long history as forest (Fig. 2).

**Figure 3:**
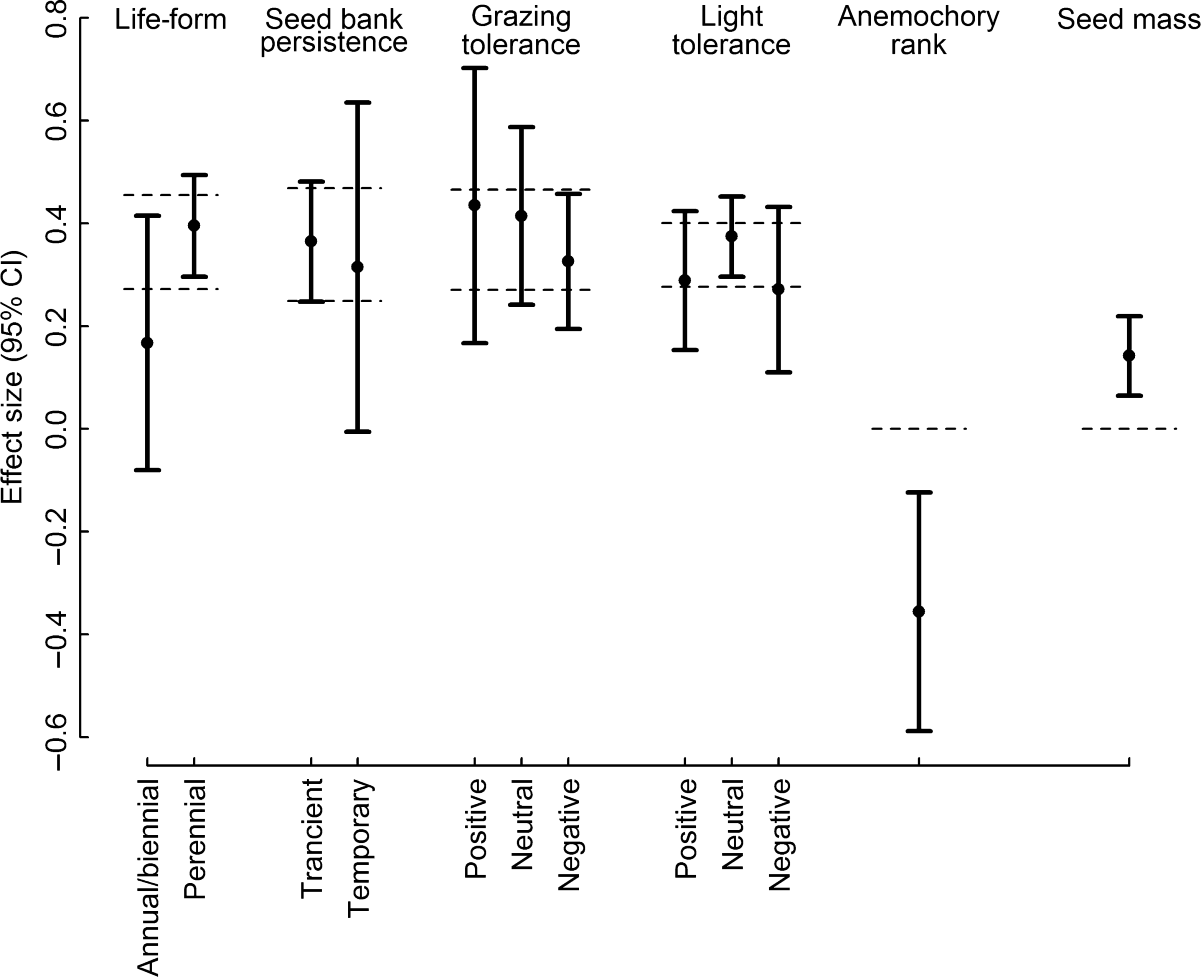
Random effects meta-regression analyzing the effect of historical land use (meadow vs. forest) on clear-cut floras using traits as explanatory variables. The area between the dotted vertical lines represents the 95% confidence interval (CI 95%) for the community weighted average and the error bars the CI 95% of each trait. Error bars above the upper CI of the community weighted average indicate a preference toward clear-cuts with history as meadow compared to forest and *vice versa*. An error bar overlapping the area between the dotted lines by less than half corresponds to *P* < 0.05.

There was significantly higher seed mass and lower wind dispersal for plants in clear-cuts with a history as meadow, whereas there was no significant effect of the other four traits analyzed in the meta-regressions (Fig. 3).

## Discussion

Due to a rapid increase in the intensity of land use during the past century, the once-widespread seminatural grasslands in central and northern Europe have suffered severe losses and fragmentation. However, in contrast to the ecological theory (MacArthur and Wilson 1967; Hanski 1994), few negative effects have been found on the grassland flora (e.g., Helm et al. 2006). The reason for this has been explained as being the slow response of many plant species to habitat degradation and fragmentation, helping them to transcend periods of suboptimal environmental conditions (Eriksson 1996; Lindborg and Eriksson 2004; Johansson et al. 2011). In fact, in line with our hypothesis, we found a legacy of grassland management in clear-cut floras at least 80 years after their conversion to coniferous forest. On average, clear-cuts with a history as meadow in the 1870s had a 36% higher plant species richness and a 35% higher number of grassland indicator species compared to clear-cuts managed as forest at that time. The difference was surprisingly large given that the area comprised by historic meadow was, on average, less than half of the total clear-cut area. Our results corroborate other studies showing clear signs of historical land use in present-day flora (Eriksson et al. 2002; Gustavsson et al. 2007) as well as in other taxa such as butterflies (Ibbe et al. 2011). Several mechanisms may explain the differences in plant richness in this study.

Seed size is positively related to seedling competitive ability and is considered an important factor shaping the patterns of abundance and dynamics of plants in species-rich seminatural grasslands, where the level of disturbance and the competition for space and resources are high (Eriksson and Jakobsson 1998; Jakobsson and Eriksson 2000). Large-seeded species were therefore expected to be proportionally more abundant in meadows compared to, for example, forests. Additionally, if meadow plant communities form remnant populations, this would be evident as a distinction in seed size between clear-cuts based on their land-use history. In fact, seed mass was larger in clear-cuts with a history as meadow compared to forest. Moreover, the generally lower anemochory in the former meadows suggests that the plants there rely on dispersal strategies other than wind. Low (wind) dispersal ability is associated with species' above-ground persistence (Ozinga et al. 2007) and a delayed response to habitat change (Kimberley et al. 2013). Thus, in explaining the positive effect of previous grassland management, the above results support our first hypothesized scenario that the plants never disappeared after afforestation but have survived as remnant populations, while refuting the second scenario that the plants have emigrated from neighboring grasslands post logging. However, in the presence of remnant populations, it was anticipated that clear-cuts with meadow history would have more perennial species and species adapted to grazed and sunlit habitats, which could not be confirmed. Neither was there an effect of the soil seed bank attributes of species, thus refuting the third scenario suggesting that the plants have persisted in the soil as seeds and emerged after clear-cutting. The rejection of the third scenario was strengthened by the fact that large seeds are less likely to be incorporated into the soil than smaller seeds (Thompson et al. 1993), although no correlation between seed mass and seed bank persistence was found for the species involved in this study. Moreover, Bossuyt et al. (2006) found that the density of seeds in the seed bank decreased with increasing time since abandonment for species associated with calcareous grasslands and concluded that restoration of grassland plant communities cannot rely on germination of target species from the seed bank alone.

Present-day grassland plant communities are often reflected in the historical configuration of habitat (Krauss et al. 2010). Based on the present study, it may also be that the loss and fragmentation of grasslands are overestimated, especially in forested landscapes, because many plant species remain in areas not conventionally classified as grassland habitats (Jonason et al. 2010). For example, Lindborg et al. (2014) found that plant species richness in midfield islets and road verges declined with the distance to the nearest seminatural grassland but only in the agriculturally dominated landscape and not in the landscape dominated by commercial forests, indicating that forests contain overlooked source populations that contribute to meta-population persistence. Moreover, former arable fields that had been grazed for up to 18 years had a 35% higher plant richness and 91% more species classified as grassland specialists if embedded in forest in contrast to arable land (Cousins and Aggemyr 2008), and plant species richness in seminatural grassland is higher if the matrix land use consists of forest instead of arable land (Söderström et al. 2001; Öckinger et al. 2012). The above results further support our scenario that the plants have persisted as remnant populations after afforestation.

## Conclusions

Protecting remaining grassland fragments is of the highest conservation concern because these have been developed over hundreds of years of extensive management and cannot be created elsewhere within a reasonable time frame. Unfortunately, given the vast literature documenting the extinction debts of grassland plant communities (e.g., Helm et al. 2006; Krauss et al. 2010), protecting only the fragments will not be enough. In a metapopulation context (Hanski and Ovaskainen 2002), the only way to reduce existing extinction debts is to increase grassland area and connectivity. We conclude that restoration of forests with a history of grassland management has great potential to be successful. First, there is a baseline flora in place that, despite a long period under harsh environmental conditions, is superior to that in clear-cuts with a forest history. Second, in many regions, seminatural grasslands accounted for more than half of the land use in Sweden in the 1800s (Dahlström et al. 2006), which offers opportunities for conservation initiatives at large scales. For example, by introducing grazing animals, lingering reforestation or planting deciduous instead of coniferous trees, the plant populations would be given a chance to recover. Third, the record of old land-use maps available over large parts of Sweden makes it possible to trace the exact locations of historical grasslands in the landscape. However, the time span to act is narrow. Swedish forests have in line with the introduction of modern forest management practices transitioned into dense, even-aged stands hostile to grassland plants (Niklasson and Granström 2000; Axelsson and Östlund 2001), and the conditions for the survival of remnant populations can be assumed to have worsened considerably. Finally, historical maps with similar content also exist for other European countries where grassland management once dominated the landscape (Petit and Lambin 2002). Thus, if the land-use legacy found here in production forest clear-cuts is a general phenomenon, historical maps may also contribute to increased restoration and conservation of grassland habitats outside Sweden.

## Supporting Information

**Appendix S1.** Descriptions of the traits used in the study.

**Appendix S2.** A complete species list.
