# Supplementary material for: Vegetation in clear-cuts depends on previous land use: a century-old grassland legacy
Source: Ecol Evol. 2014 Oct 24;4(22):4287–95. doi: 10.1002/ece3.1288 (PMC4267867; doi:10.1002/ece3.1288)
Supplement: Supplementary file 1 — Appendix S1. Descriptions of the traits used in the study. [file ece30004-4287-SD1.docx]

Appendix 1. Description of traits used in the study.

| **Trait** | **Trait description** | **Data availability** | **Sources** |
| --- | --- | --- | --- |
| Life span | Plant life span (annual/biennial or perennial) | 100% | www.lundsbotaniska.se |
| Diaspore mass | Mass of one diaspore (mg) including all appendages | 94% | Hintze et al. 2013; Kleyer et al. 2008 |
| Seed bank persistence | The persistence of the seeds in the soil seed bank; short-term (<25 years) or permanent (>25 years) | 72% | www.lundsbotaniska.se |
| Anemochory index | A ranking index on the adaptation to anemochory based on the terminal velocity (i.e. the maximum speed (m/s) of a falling diaspore in still air) of c. 2700 European plant species. | 92% | Hintze et al. 2013 |
| Grazing tolerance | The extent to which the plants are adapted to grazing and mowing, categorized as high, neutral, or low | 88% | www.lundsbotaniska.se |
| Light tolerance | Ellenberg Indicator Value for light simplified into three categories; high, neutral or low | 92% | www.lundsbotaniska.se |
